# Visual- spatial capacity: gender and sport differences in young volleyball and tennis athletes and non-athletes

**DOI:** 10.1186/1756-0500-7-57

**Published:** 2014-01-21

**Authors:** Angela Notarnicola, Giuseppe Maccagnano, Vito Pesce, Silvio Tafuri, Grazia Novielli, Biagio Moretti

**Affiliations:** 1 Course of Motor and Sports Sciences, Faculty of Medicine and Surgery, University of Study of Bari, Lungomare Starita 1, 70123 Bari, Italy; 2 Orthopedics Section, Department of Neuroscience and Organs of Sense, Faculty of Medicine and Surgery, University of Study of Bari, General Hospital, Piazza Giulio Cesare 11, 70124 Bari, Italy; 3 Department of Biomedical Sciences and Human Oncology, Faculty of Medicine and Surgery, University of Study of Bari, General Hospital, Piazza Giulio Cesare 11, 70124 Bari, Italy

**Keywords:** Visual-spatial ability, Gender difference, Volleyball, Tennis

## Abstract

**Background:**

In the general population visual-spatial ability is better in males, due to the influence of biological and socio-cultural factors. We know that sport activity improves motor skills. The aim of this work is to determine if these gender differences exist in young athletes. The orientation test described by Terzi and standardized by Cesaroni, used to measure spatial ability, was carried out on 60 volleyball or 60 tennis athletes as well as on 60 non-sporting subjects.

**Results:**

The data analysis revealed a worse performance for non-athletes in comparison with athletes in both components of test (p < 0.0001; p = 0.04), with no differences between the volleyball and tennis groups. As far as gender comparison is concerned, as expected in the non- sport group the males presented better values (p < 0.001; p = 0.006). However in both sports groups there weren’t any gender differences in either part of the test (p = 0.18; p = 0.056).

**Conclusions:**

These results confirm that during athletic preparation in volleyball and tennis the specific training is able to develop spatial ability. Besides, boys and girls have similar performance demands and training experience. It appears that this specific training could be responsible for modifying gender differences in performance of spatial ability during adolescence.

## Background

Motor skills express the possibility to move in a way that each person is able [1]. It’s possible to identify conditional and coordinative abilities. Both depend on hereditary traits, but are also developed through motor activity. They are stable and long lasting and they are responsible for sport performance.

An important coordinative ability is visual spatial skill [2]. This ability is to prevent us getting lost and being able to read or build a map of the surrounding space. It is achieved and maintained by a complex set of sensory motor control systems that include: sensory input from vision, proprioception, and the vestibular system. To determine this ability, intrinsic individual components and environmental factors intervene [3].

An important example of intrinsic characteristics are gender differences. Men show better spatial ability than women [4,5]. Different theories on anatomical evolution have been used to justify these differences. It has been suggested that these gender differences arose due to gender roles in the early human ages, when men went out hunting, while women stayed with the children while gathering food or carrying out manual labour [6]. Another explanation for these sex differences focuses on hormones. Testosterone in males leads to better spatial performance, while oestrogen in females leads to a reduction in their mobility in order to devote themselves to parental care [7]. Other researchers have reported that males tend to have larger brain volume, while the grey-to-white ratio tends to be greater in females [8-10].

As far as environmental stimulation is concerned, sport plays an important role [11]. Motor response to various situation needs adequate spatial evaluation. In general, tactical preparation allows sports men to develop spatial skills, which are divided into 5 types: technical (minimal distance between striker and defender), tactical (space in the defense), projective (how much place the player has to move) dynamic (the real field maybe smaller then player imagines) and topological (space an ether side, behind and in front, above and below the player) [11]. These different perceptions justify how sport activity could improve attention levels and visual-spatial ability. The aim of this work is to verify if by practicing sport there are still gender differences between young athletes in two different sports (volleyball and tennis) compared to a non-sport activity control group.

## Methods

We set up a clinical observational study designed to recruit volunteer athletes to undergo a motor skill test, halfway through the tournament season. The control group was formed by non- athletes. Ethical approval was given by the local Ethics Committee of Bari University General Hospital and written informed consent was obtained from the parent or guardian of each participant.

In January and February 2012 we recruited young people who took part in semi-professional volleyball (1^st^ group, 60 subjects) and a tennis group (2^nd^, 60 subjects) and who reported no historical speciality in any sport/exercise and were sedentary at the time of the study (3^rd^ group, 60 subjects). Each group was formed by 30 males and 30 females. The inclusion criteria of subjects was to be between age 11–14 with no musculoskeletal or neurological disorders, nor were they any taking any medication that could affect cognitive functions. For the two sport groups another inclusion criteria was years of sport activity, of between two and four years. For each sport the subjects were recruited from the same school. The athletes had an ongoing training programme: 2 hours a day, 2 or more days a week.

**Figure 1:**
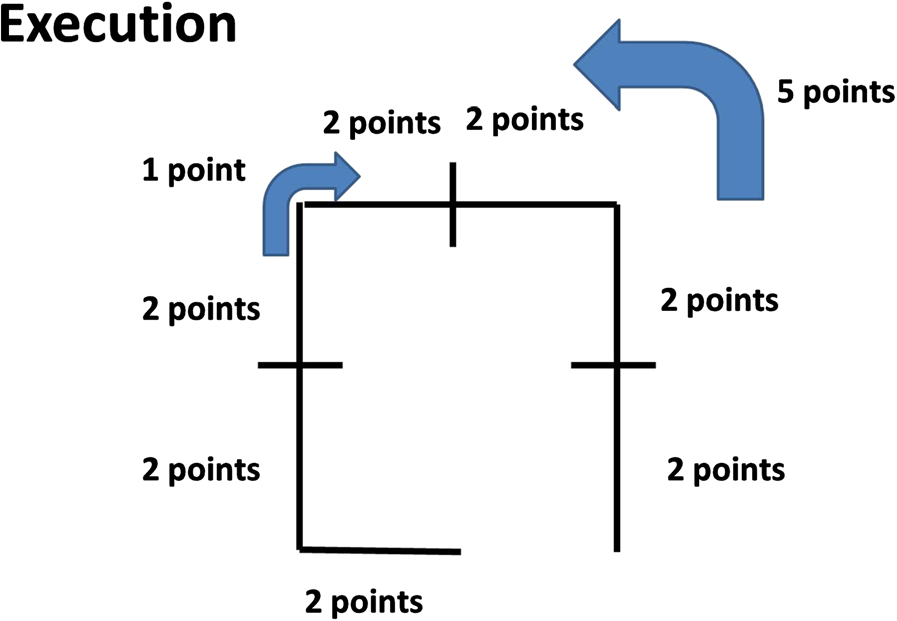
Route that the subject has to complete under vocal command during the first part of the test.

**Figure 2:**
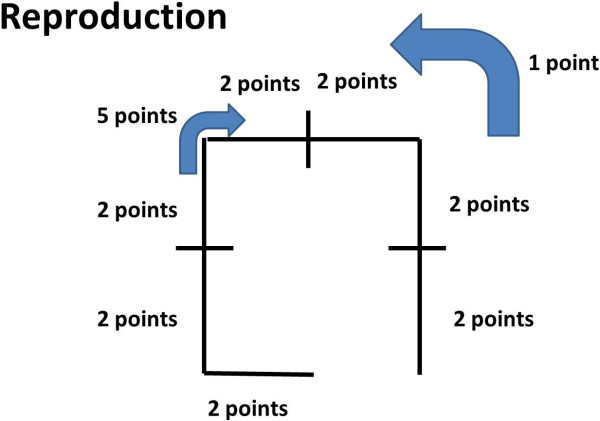
Route that the subject should be able to reproduce on paper during the second part of the test.

The Applied Test is described by Terzi and standardized by Cesaroni, useful to study these motor skill [12]. The test was carried out before a training session on the sport subjects. The test was carried out by the same researcher (AN) who has 7 years of experience in research and studies on motor methodology. The test is composed of two parts. In both phases of the test during the execution and production a point system was assigned for each right command (Table 1 and Figures 1 and 2).

**Table 1:** The command given during the two parts of the test and the corresponding scores

1^st^ part of the test: execution	
Command given	The blindfolded subject has to execute the following commands:
	- Take a lateral left step,
	- Take two steps forward,
	- Turn right 90°,
	- Take two steps forward,
	- Take a lateral right step,
	- Go back to the starting point.
Point assigned	- Steps forward: 2 points
	- Lateral steps: 2 points
	- Right turn: 1 point
	- Go back correctly: 5 points
	Total score: 20 points
2^nd^ part of the test: reproduction	
Command given	We ask to subject to design the route.
Point assigned	- Lateral step: 2 points
	- Steps forward: 2 points
	- Right turn 90°: 5 points
	- Right lateral steps: 2 points
	- Go back to the starting point: 1 point
	Total score: 20 points

• During the 1^st^ part, execution, the blindfolded subject has to carry out a command given by an examiner, memorize them and imagine the route taken to get back to the starting point. The route is square. The last command, to go back to the starting point, tests orientation skills.

• During the 2^nd^ part, reproduction, the subject has to reproduce on paper the route taken.

For each subject recruited we completed a file in which we reported the epidemiological data as well as the test results. The forms were entered into a database using FileMaker Pro software. We used the STATA MP11 software to analyse the data. Quantitative variables were expressed as mean and standard deviation. We used the t-student test for independent samples to compare the mean sex and sport activity. Furthermore, we performed multivariable regression models. For every test we considered a value of p < 0.05 to be statistically significant.

## Results

### Epidemiological data

The demographic data of each group is shown in Table 2. The groups are comparable for epidemiological characteristics. All subjects had normal or corrected-to-normal visual accuracy and were right-handed. The results of both parts of the test are shown in Table 3 for each group.

**Table 2:** Age and years of sport activity, for each study group

**Group**	**Age (years)**	**Years of sport activity**
Tennis players	12.4 ± 0.7	2,5 ± 0.8
Volleyball players	12.6 ± 0.8	2.4 ± 0.7
Non-sport subjects	12.5 ± 0.8	NA
p-value	0.69	0.69

The data is expressed by average and standard deviation. The statistical comparison between groups is expressed by p value.

**Table 3:** Average values and standard deviation of execution and repetition points for group

**Group**	**1**^**st **^**part of the test: execution**	**2**^**nd **^**part of the test: reproduction**
Tennis players	19.1 ± 1.7	13.1 ± 6.4
Volleyball players	19.0 ± 1.9	10.7 ± 7.1
Non-sport subjects	13.0 ± 1.6	9.7 ± 2.4
F	120.8	2.91
P-value	<0.001	0.06

We performed a statistical comparison between three groups.

### Results of comparison between groups

The sport subjects (volleyball and tennis players) obtained the average value of 19.1 ± 1.8 during the execution and 11.9 ± 6.8 during the reproduction. These results are statistically better than the non-sport subjects considering test execution (t = 15.2; p < 0.0001) and reproduction (t = 1.8; p = 0.04). There weren’t any statistically significant differences in the results of both parts of test between the two sport groups (respectively t = 0.21, p = 0.41; t = 1.37; p = 0.09).

### Gender comparison

In the control group the males presented statistically better values (execution = 14.1 ± 0.7; reproduction = 11 ± 2.8) compared to the females (execution = 11.9 ± 1.6; reproduction = 8.3 ± 0.61) for each part of the test (t = 4.9; p < 0.001; t = 3.63; p = 0.006).

In the sport group there were no statistically significant differences in execution (t = 0.93; p = 0.18), while we verified statistically better results (t = 1.6; p = 0.056) in the females (13.3 ± 6.9) compared to the males (10.5 ± 6.5) during reproduction.

### Multivariate analysis

The multivariable regression models highlights how the values are influenced by gender (t = 2.88; p = 0.005), belonging to the volleyball group (t = 7.78; p < 0.0001) or the tennis group (t = 7.27; p < 0.0001). Any variables included in the model influence the values obtained during the reproduction.

## Discussion

The results of this work allow us to study gender differences in visual-spatial skills during volleyball and tennis activity. Using the Terzi test standardized by Cesaroni we were able to verify two components of spatial ability: spatial orientation and memory. The test is therefore composed of two parts. During the first part, the execution, the subject has to follow vocal command, complete the route and go back to the starting point. The second part of the test, the reproduction, required the subject to reproduce on paper the route taken. Through this test we are able to evaluate spatial capacity.

As expected from literature, we found better results in the sport group [13,14]. This new data has not been studied until now. The same results were found in both sports. In sport this data is linked to the perception of space. First of all in two elements: information stored by different technicians and specific techniques of spatial ability [11]. In particular, an athlete has to memorize all the necessary information and keep it in mind even when it is not in his field of vision. This also brings improvement in motor skills such as anticipation, reaction, balance, motor transformation, kinesthetic differentiation, etc. and so improves sporting ability. In volleyball and tennis we found similar aspects: the ball, the net, opponents, teammates, lines and the court. In both sport the athlete must constantly be aware of the position of the ball (main target), but also keep an eye on the opponents’ position, teammates and the lines of the court (secondary target). As far as basic techniques are concerned we must remember that a game can be "invasional" (football, basketball, handball, rugby, hockey, etc.) or "cross-reference" (volleyball, tennis, squash, etc.) [11]. In both sports analyzing the difficulty of the game is similar. The field is divided into two parts separated by a net. During the game a spatial limit is necessary to keep all the players on their side of the field so spatial perception is linked to the ball movements on the opponents’ side, while trying to score a point as well as stopping the opponents from scoring a point on their own side.

In the control group we verified the presence of gender differences for both parts of the test. This result is similar to literature where we can see better results among man rather than women for different visual-spatial tasks: spatial perception, spatial visualization, mental turn, spatial-temporal ability, generation and maintenance mental imagines and animation [15]. This justifies better orientation in men than women because they learn routes more quickly and are better at estimating distances thanks to high visual ability. This ability comes from a mental rotation strategy [16]. Women use a strategy based on route imagination using reference points (signs, etc.) [16]. In the test the subject is blindfolded and so has no reference points which accounts for the bad results among the women in the control group.

We found no statistically significant gender differences in either sport group. These results are linked to sports’ history and male prevalence in teaching and practicing [17,18]*,* while in the last years women have begun to learn the same male tactical schemes [19].

In volleyball and tennis boys and girls have similar demands in performance and training experience. According to the data found in our study we hypothesize that spatial ability is influenced by sport activity and the latter could decrease gender differences.

Another hypothesis could be hormonal modulation which is induced by the sport practice. Biological hypotheses are based on the assumption that sexual hormones influence cognitive development. In fact, hormone manipulation affects not only sexual behavior, but also some aspects of cognition, in particular spatial ability [20]. Physiologically male gender is characterized by high circulating androgen levels which are responsible for better male orientation [14]. We have to consider that sport activity could increase circulating androgens levels. Previous studies have verified that in some sport which are medium impact sports, such as baseball, swimming, or track, spatial orientation is similar in males and females. In other sports, such as basketball, which are higher impact, females show an advantage over males. It was verified that androgens could improve spatial ability in women, but could inhibit it in men [14]. These results are linked to a physical level of impact and consequently to hormonal instability could justify different capabilities found in the sporting population compared to the general population [14]. In our case in consideration of athletes agonistic levels the impact of the sport was medium. Even though we didn’t measure hormonal levels, we can hypothesize that there was a medium hormonal stimulation in both genders which justify the similar results between the males and females.

Our finding of athletes’ superiority in spatial memory working is in accordance with literature [21-23].

The training improves this capacity, which is a fundamental element for high profit for the sport activities [22,23]. Magnetic resonance images show that athletes have a significantly increased cortical thickness in specific areas of brain involving visual system capacities [24]. On the other hand, we did not find any differences between tennis and volleyball athletes, neither between male and female athletes. These results are in discordance with a recent meta-analysis, in which the researchers examined how cognitive capacity could be influenced by the sport type and by sex [22]. The authors found that athletes from interceptive sport types (as tennis) and males performed better. However, previous researchers have noted some weaknesses in these studies such as small sample sizes and methodological heterogeneity [22]. Moreover, the authors pointed out that there are more studies involving male than female athletes and more work related to interceptive than strategic sports (as volleyball) or static sports (as running) [22,25].

On the basis of our preliminary results we hypothesize that in the athletes the experience-dependent learning and brain plasticity could level the differences of cognitive skills correlated to the sport type and gender. Further studies with large sample sizes could verify this assumption.

The weak points of the study are the absence of prospective design that could have allowed us to follow up possible variations of motor skills during the agonistic season. The observation of volleyball and tennis athletes needs to be expanded to athletes in other different sports, in particular during closed skill sport activity such as swimming, running, invasive sports such as football and basketball. In following works it could be useful to analyze gender influence on the other coordinative motor skills. The test group is restricted to 11 to 14 years of age. Since this is the age group where there are the most hormonal changes in the adolescents and where the maturation rate is the most different between boys and girls, we need to consider the difficulty to establish the origin of the differences observed in this measure between sexes [26].

Despite these limitations, our study has the merit of being the first to examine the sex-based differences for visuo-spatial ability in volleyball and tennis.

## Conclusions

This data supports the idea that sport activity reduces gender differences in spatial ability during the adolescence. This discovery could give us clues to be used in teaching. As a matter of fact, not all children take part in sport which allows them to increase visuo-spatial motor skills. For this reason we could think, for example, of suggesting traditional games (cops and robbers, tag, hopscotch, etc.) which help to develop spatial ability in children.

## Availability of supporting data

The data are deposited at University of Bari, Course of Motor and Sports Sciences (Grazia Novielli’s thesis of degree).

## Competing interests

The authors declare that they have no competing interests.

## Authors’ contributions

AN, GM and BM drafted of the manuscript and reviewed the literature. AN, GN and VP conceived the study, participated in its coordination and in the acquisition of the data. ST gave substantial contributions to statistical analysis and interpretation of data. All authors read and approved the final manuscript.

## References

[B1] Schmidt RA Wrisberg CA Motor Learning and Performance 2008 Champaign, IL: Human Kinetics

[B2] Wolbers T Hegarty M What determines our navigational abilities? Trends Cogn Sci 2010 14 3 138 146 10.1016/j.tics.2010.01.001 20138795

[B3] Vona G Massidda M Cireddu MI Calò CM Genetics and sport performance Ital J Sport Sci 2005 12 105 115

[B4] Hall JA Kimura D Sexual orientation and performance on sexually dimorphic motor tasks Arch Sex Behav 1995 24 395 407 10.1007/BF01541855 7661655

[B5] Watson NV Kimura D Right-hand superiority for throwing but not for intercepting Neuropsychologia 1989 27 1399 1414 10.1016/0028-3932(89)90133-4 2615939

[B6] Kimura D Human sex differences in cognition, fact, not predicament Sex Evol Gend 2004 6 45 53 10.1080/14616660410001733597

[B7] Healy SD Braham SR Braithwaite VA Spatial working memory in rats: no differences between the sexes Proc Biol Sci 1999 266 1435 2303 2308 10.1098/rspb.1999.0923 10629980 PMC1690445

[B8] Allen JS Damasio H Grabowski TJ Bruss J Zhang W Sexual dimorphism and asymmetries in the gray-white composition of the human cerebrum Neuroimage 2003 18 880 894 10.1016/S1053-8119(03)00034-X 12725764

[B9] Gur RC Gunning-Dixon F Bilker WB Gur RE Sex differences in temporo-limbic and frontal brain volumes of healthy adults Cereb Cortex 2002 12 998 1003 10.1093/cercor/12.9.998 12183399

[B10] Shikhman M Age, Gender, General Intelligence and Educational Level Influences on Working Memory 2007 1 City University of New York, USA: ProQuest Editor 107

[B11] Ceciliani A Elementi di didattica degli giochi sportive: l’allievo e lo spazio-tempo SdS/Rivista di cultura sportiva 2005 XXIII 60–61 61 68

[B12] Brugnoni G Alpini D Medicina fisica e riabilitativa nei disturbi di equilibrio 2007 Italy: Publisher Springer-Verlag

[B13] Lord T Leonard B Comparing scores on spatial-perception tests for intercollegiate athletes and nonathletes Percept Mot Skills 1997 84 1 299 306 10.2466/pms.1997.84.1.299 9132723

[B14] Lord TR Garrison J Comparing spatial abilities of collegiate athletes in different sports Percept Mot Skills 1998 86 3 Pt 1 1016 8 9656301 10.2466/pms.1998.86.3.1016

[B15] Silverman I Choi J Peters M The hunter/gatherer theory of sex differences in spatial abilities: data from 40 countries Arch Sexual Behav 2007 36 261 268 10.1007/s10508-006-9168-6 17351740

[B16] Ward S Newcombe N Overton W Turn left at the church, or three miles north: a study of direction giving and sex differences Environ Behav 1986 18 192 213 10.1177/0013916586182003

[B17] Chance JE Goldstein AG Internal-external control of reinforcement embedded-figures performance Percept Psychophys 1971 9 33 34 10.3758/BF03213024

[B18] Connor JM Serbin LA Schakman M Sex differences in children’s responses to training in a visual-spatial test Dev Psychol 1977 13 293 294

[B19] Ryan JP Atkinson TM Dunham KT Sports-related and gender differences on neuropsychological measures of frontal lobe functioning Clin J Sport Med 2004 14 18 24 10.1097/00042752-200401000-00004 14712162

[B20] Williams CL Barnett AM Meck WH Organizational effects of early gonadal secretions on sexual differentiation in spatial memory Behav Neurosci 1990 104 1 84 97 2317288 10.1037//0735-7044.104.1.84

[B21] Mann DT Williams AM Ward P Janelle CM Perceptual-cognitive expertise in sport: a meta-analysis J Sport Exercise Psy 2007 29 4 457 478 10.1123/jsep.29.4.457 17968048

[B22] Voss M Kramer AF Prakash RS Roberts B Basak C Are expert athletes "expert" in the cognitive laboratory? A meta-analytic review of cognition and sport expertise Appl Cogn Psychol 2009 24 812 26

[B23] Faubert J Professional athletes have extraordinary skills for rapidly learning complex and neutral dynamic visual scenes Sci Rep 2013 3 1154 23378899 10.1038/srep01154 PMC3560394

[B24] Wei G Zhang Y Jiang T Luo J Increased cortical thickness in sports experts: a comparison of diving players with the controls PLoS One 2011 6 2 e17112 10.1371/journal.pone.0017112 21359177 PMC3040218

[B25] Lum J Enns JT Pratt J Visual orienting in college athletes: explorations of athlete type and gender Res Q Exerc Sport 2002 73 2 156 167 10.1080/02701367.2002.10609004 12092890

[B26] Sisk CL Zehr JL Pubertal hormones organize the adolescent brain and behavior Front Neuroendocrinol 2005 26 3–4 163 74 16309736 10.1016/j.yfrne.2005.10.003

